# Isolation, molecular identification, and antifungal susceptibility profiles of vaginal isolates of *Candida* species

**Published:** 2016-12

**Authors:** Ali Rezaei-Matehkolaei, Shokoofe Shafiei, Ali Zarei-Mahmoudabadi

**Affiliations:** 1 Infectious and Tropical Diseases Research Center, Health Research Institute, Ahvaz Jundishapur University of Medical Sciences, Ahvaz, Iran; 2 Department of Medical Mycology, School of Medicine, Ahvaz Jundishapur University of Medical Sciences, Ahvaz, Iran

**Keywords:** Caspofungin, Fluconazole, Clotrimazole, *Candida* vaginitis, *Candida albicans*

## Abstract

**Background and Objectives:**

Vulvovaginal candidiasis is a common fungal infection among women during reproductive ages. Although, *Candida albicans* is accounted as the main etiologic agent of vaginitis, non-*albicans* species have arisen during last years. Resistant to antifungal drugs especially, fluconazole has been more reported by researchers from around the World. The aims of this study were to determine the prevalence of vulvovaginal candidiasis among suspected patients with vaginitis, the frequency of *Candida* species, and the susceptibility profiles of isolates to caspofungin, fluconazole and clotrimazole.

**Materials and Methods:**

One hundred and twenty suspected women with vaginitis were examined by specialist physician and sampled using moisture swabs. Swabs were inoculated on CHROMagar *Candida* plates, incubated at 35°C and detected all isolated *Candida* species using morphological, microcopy and molecular methods. The antifungal susceptibility tests with caspofungin, fluconazole and clotrimazole were applied using microdilution and Resazurin dye methods against all isolated yeasts.

**Results:**

The cultures were positive for 34(28.3%) samples and three *Candida* species including; *C. albicans* (88.2%), *C. glabrata* (8.8%) and *C. kefyr* (2.9%). Our study shows that only one isolate of *C. albicans* was resistant to caspofungin at the concentration of 2 μg/ml after 24h incubation that increased to 2 isolates after 48h incubation. All isolates were sensitive to fluconazole at the MIC ranges of 1-0.25 μg/ml, while 88.2% of them were inhibited at 0.25 μg/mL of clotrimazole. *Candida albicans* remains the most common agent of fungal vaginitis.

**Conclusion:**

Although all of *Candida* isolates were susceptible to fluconazole *in vitro*, it should be used with caution for empirical therapy due to more resistant rates in clinic. In addition, due to valuable sensitivity of all tested strains to caspofungin, it potentially can be presented as the first line therapy for *Candida* vaginitis.

## INTRODUCTION

Vaginitis or vulvovaginal candidiasis is a common fungal infection among women during reproductive ages with worldwide distribution (1). In addition, immunocompromised patients (especially HIV positive) are usually associated with chronic or persistent forms of vaginitis (2). External factors such as, IUD using, antibacterial and corticosteroid therapy, type II diabetes, and psychosocial stress are important risk factors for *Candida* vaginitis (3–5). The frequency of disease varies in reports from 62.1% (6), 49% (2) and, 28.2% (7). Although, several antifungal drugs were applied for vulvovaginal candidiasis, the rate of disease did not decrease during several last decades. Many reports have shown that 75% of healthy women experiences at least one symptomatic vulvovaginal candidiasis during their lifetime (8). On the other hand, some reports indicated that vulvovaginal candidiasis is an important problem among pregnant women (9). In these cases, neonates (particularly, low birth weight and prematurity) are usually exposed to systemic candidiasis after delivery (9).

*Candida albicans* was accounted as the first vulvovaginal candidiasis causative agent followed by *C. glabrata* and *C. tropicalis* (10–12). Others non-*albicans* species included; *C. africana* (13), *C. dubliniensis* (13), *C. parapsilosis* (14), *C. guilliermondii* (3), *C. nivariensis* (15), *C. bracarensis* (15), *C. kefyr* (4), *Saccharomyces cerevisiae* (16), *C. pintolopesii* (7) and *C. krusei* (17). An increasing from 10% to 20% was reported from 1970s to 1980s for *C. glabrata* and *C. tropicalis* as *Candida* vaginitis agents (3). Clotrimazole and fluconazole are two azole antifungal drugs that are usually prescribed for *Candida* vaginitis treatment. Although, fluconazole was routinely used for the treatment of vaginal candidiasis, the rate of resistance varies in different studies, 11.8% to 94% (6, 18, 19). The sensitivity of *C. tropicalis* and *C. glabrata* to miconazole is 10 times less than *C. albicans* (3).

Some studies have shown that vaginitis due to *C. krusei* is resistant to fluconazole (5). Furthermore, clotrimazole resistant strains (1.8%) were also reported (18). Caspofungin is a new echinocandin antifungal that has been used during last two decades for invasive fungal infection in immunocompromised patients (20) and, transplant recipients (21). It inhibits cell wall 1,3-β-D-glucan synthesis (22) and there are few available reports associated with resistance to this antifungal. Caspofungin has an excellent safety profile with good efficacy for antifungal therapy among patients with invasive aspergillosis and candidiasis (23, 24). On the other hand, few reports evaluated the efficacy of caspofungin against vaginal *Candida* isolates *in vitro*. The aims of this study were to determine the prevalence of vulvovaginal candidiasis among patients suspected to vaginitis, the frequency of *Candida* species, and the susceptibility profiles of isolates to caspofungin, fluconazole and clotrimazole.

## MATERIALS AND METHODS

### Patients and sampling

This project was approved in the ethical committee of Ahvaz Jundishapur University of Medical Sciences (IR.AJUMS. REC.1394.285). All patients were signed the consent form before sampling. Demographic details and pre-disposing factors for each patient were recorded. In the present study 120 suspected women with vaginitis were examined by the specialist physician and sampled using moisture swabs. The presence of signs and symptoms including, erythema, edema, excoriation, discharge, pruritus, soreness, and burning were defined as common signs and symptoms of *Candida* vaginitis. Vaginal swabs were inoculated on CHROMagar *Candida* (CHROMagar Candida, France) plates, incubated at 35°C for 4 days, aerobically. All plates were evaluated for the yeast growth and colony colour every day. Direct microscopy slides were prepared from each colony for yeast confirmation. All yeast isolates were duplicately subcultured on Sabouraud dextrose agar (SDA, Merck, Germany) plates and kept at room temperature for future mycological analyses.

### Laboratory procedures

After isolation from CHROMagar *Candida* medium, all yeast strains were subsequently identified using classical mycological tests for the genus *Candida*, such as germ tube formation on fresh human serum at 37°C, microscopic morphology on cornmeal agar (High Media, India) with 1% Tween 80 (Merck, Germany) and growth at 42–45°C for 24–48h (25).

### PCR-RFLP method

For molecular identification, a loopful of fresh colony of each strain was suspended in a 1.5 ml microtube contained 100 μl of deionized distilled water and heated at 100°C for 10 minutes. Then, microtubes were centrifuged at 4000 g for 10 minutes. The supernatants were transferred to a new microtube and kept at −20°C as DNA. The nuclear ribosomal ITS1-5.8S-ITS2 regions of the strains were amplified through PCR from extracted DNAs using the ITS1/ITS4 primer pair (26). The obtained products were then subjected to restriction analysis with *Msp*I enzyme (Thermo Fisher Scientific, Waltham, MA, USA). For final identification of the isolates, the restriction products were electrophoresed through 2% agarose gel, and size of digested fragments were compared with those determined in the previous report (27).

### Antifungal stock solutions

A stock solution of antifungal drugs including; caspofungin (Sigma-Aldrich, Germany) 1.25 mg/ml, fluconazole (Serva, USA) 32 mg/ml and clotrimazole (Sigma-Aldrich, Germany) 32 mg/ml was prepared in dimethyl sulf-oxide (DMSO, Fluka, Germany). Antifungal stocks were kept at −20°C until use.

### RPMI 1640 containing Resazurin

The 0.01g of Resazurin (Sigma-Aldrich, Germany) was completely dissolved in 100 ml of distilled water and then sterilized using a syringe filter (0.45μm). Stock solution was kept in a brown bottle at 4°C until use.

### Standard suspension preparation

A suspension of overnight culture of each isolate on SDA incubated at 35°C was prepared in sterile PBS. Suspensions were adjusted to 0.5 McFarland standard (1.5 × 10^8^ CFU/ml) using a spectrophotometer. The suspensions were diluted as 1:50 with RPMI 1640 containing Resazurin in sterile condition.

### *In vitro* antifungal susceptibility test

The antifungal susceptibility tests with caspofungin, fluconazole and clotrimazole were applied using microdilution and Resazurin dye methods against all vaginal isolates (28, 29). Briefly, 100 μl of serial dilution of each antifungal and 100 μl of standard suspension were added to microplate wells. Serial dilutions were as 4 to 0.03125μg/ml for caspofungin, 32 to 0.25μg/ml for fluconazole and 16 to 0.125μg/ml for clotrimazole. Positive and negative controls were also included in test as RPMI 1640 with and without fungal suspension, respectively. The minimum inhibitory concentration (MIC) ranges and geometric means (GM) were determined after 24 and 48h of incubation. Furthermore, the MIC_50_ and MIC_90_ values were calculated for those species with 10 or more isolates (25).

## RESULTS

### Demographic results

Totally 120 women suspected to vulvovaginal candidiasis were physically examined and sampled for fungal infection. Patients ages ranged from >20 to <50 years, however most of the patients were in the 41–50 years old groups. Table 1 shows the details of predisposing factor among 120 sampled patients. As shown several predisposing factors were found in 65% of sampled patients. The most frequent predisposing factor was history of vaginitis, in 54 cases out of 120 suspected patients (45%).

**Table 1.** Predisposing factors among 120 sampled patients

**Predisposing factors**	**Age groups (Year)**					**Total**
	**>20**	**21–30**	**31–40**	**41–50**	**<50**	
Contraceptive use	0(0.0%)	2(2.6%)	4(5.1%)	0(0.0%)	0 (0.0%)	6(7.7%)
Antibiotic therapy	0(0.0%)	4(5.1%)	3(3.8%)	4(5.1%)	1(1.3%)	12(15.4%)
Previous vaginitis	0(0.0%)	18(23.1%)	15(19.2%)	14(17.9%)	7(9.0%)	54(69.2%)
Diabetes	1(1.3%)	0(0.0%)	0(0.0%)	0(0.0%)	4(5.1%)	5(6.4%)
Cancer	0(0.0%)	0(0.0%)	0(0.0%)	0(0.0%)	1(1.3%)	1(1.3%)
Total	1(1.3%)	24(30.8%)	22(28.2%)	18(23.1%)	13(16.7%)	78(100%)

**Fig. 1.**
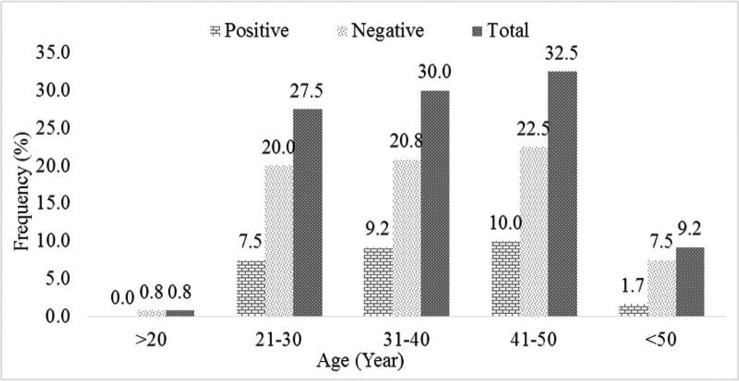
The frequency of positive and negative samples for *Candida* strains among different age groups

### Culture results

**Fig. 2.**
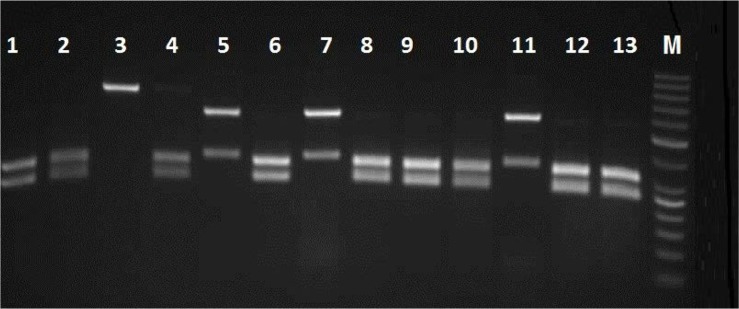
Agarose gel electrophoresis of ITS-rDNA RFLP profiles by *Msp*I for representative isolates of *C. albicans, C. glabrata* and *C. kefyr* Lanes 1, 2, 4, 6, 8, 9, 10, 12 and 13; clinical isolates of *C. albicans*, Lanes 5,7 and 11; *C. glabrata* and lane 3; *C. kefyr*, M; 50 bp DNA marker

The culture results show that 28.3% (34 of 120) of sampled patients were yielded different species of *Candida.* According to classical and molecular identification techniques, 30(88.2%) isolates of *C. albicans*, 3(8.8%) isolates of *C. glabrata* and one (2.9%) isolate of *C. kefyr* were detected. Fig. 2 shows the PCR-RFLP results for identification of some study isolates representative of three species of *Candida.*

### Susceptibility test results

Different concentrations of caspofungin, clotrimazole and fluconazole were applied against 34 isolates of *Candida* recovered from *Candida* vaginitis. As shown in the Table 2, only one isolate of *C. albicans* were resistant to caspofungin at the concentration of 2 μg/ml after 24h incubation that increased to 2 isolates after 48h incubation. MIC_50_ and MIC_90_ for all isolates, *albicans* and non-*albicans Candida* species, were found to be at 1 μg/ml. All *C. albicans* isolates were sensitive to fluconazole at the MIC ranges 1-0.25 μg/ml, while non-*albicans* species of *C. glabrata* and *C. kefyr*, were associated to 0.25 μg/ml (Table 2). The 88.2% of isolates were inhibited at 0.25 μg/ml of clotrimazole (sensitive to clotrimazole), whereas three isolates found to be dose dependent at the concentration of 0.5 μg/ml. Only one isolate (2.9%) was inhibited at 1 μg/ml of clotrimazole which considered as resistant to drug (Table 2).

**Table 2.** Geometric mean MIC, MIC range, MIC_50_ and MIC_90_ (μg/ml) of caspofungin, fluconazole and clotrimazole for 34 tested *Candida* strains

**Caspofungin (μg/ml)**		***C. albicans* (30)**		***C. glabrata* (3)**		***C. kefyr* (1)**		**Total (34)**	
			
		**24h**	**48h**	**24h**	**48h**	**24h**	**48h**	**24h**	**48h**
**R**	**2**	1	2	-	-	-	-	1	2
	**1**	24	26	-	-	-	1	24	27
**S**	**0.5**	3	2	-	-	-	-	3	2
	**0.25**	2	-	-	-	-	-	2	-
	**0.125**	-	-	3	3	1	-	4	3
**MIC**_**50**_		1	1	-	-	-	-	1	1
**MIC**_**90**_		1	1	-	-	-	-	1	1
**MIC**_**GM**_		0.87	1	-	-	-	-	0.69	0.83

**Fluconazole (μg/ml)**									
	**1**	2	5	-	-	-	-	2	5
**S**	**0.5**	7	6	-	-	-	-	7	6
	**0.25**	21	19	3	3	1	1	25	23
	**0.125**	-	-	-	-	-	-	-	-
**MIC**_**50**_		0.25	0.25	-	-	-	-	0.25	0.25
**MIC**_**90**_		0.5	1	-	-	-	-	0.5	1
**MIC**_**GM**_		0.32	0.36	-	-	-	-	0.31	0.35

**Clotrimazole (μg/ml)**									
**R**	**1**	1	3	-	-	-	-	1	3
**DD**	**0.5**	3	2	-	-	-	-	3	2
**S**	**0.25**	26	25	3	3	1	1	30	29
	**0.125**	-	-	-	-	-	-	-	-
**MIC**_**50**_		0.25	0.25	-	-	-	-	0.25	0.25
**MIC**_**90**_		0.5	0.5	-	-	-	-	0.5	0.5
**MIC**_**GM**_		0.29	0.32	-	-	-	-	0.28	0.29

MIC: Minimum Inhibitory Concentration; GM: Geometric Mean; R, Resistant; DD: Dose dependent; S, sensitive

## DISCUSSION

Vulvovaginal candidiasis is one of the most common fungal infections among adult women during bearing child period. Several authors have believed that 75% of women are affected at least once during lifetime (3, 30). Furthermore, chronic vaginitis and recurrent vulvovaginal candidiasis were more reported among several groups of women (31–34). Both forms of diseases are problematic conditions for patients. The healthy women vagina is containing several normal microflora including *C. albicans*, and patients associated factors, organism pathogenic factors and external factors are interference in involving disease. In our study, the overall prevalence of *Candida* vaginitis was found to be 28.3% which is similar to Fornari et al. (35), Rasti et al. (9), and Hedayati et al. (7), while it is in contrast to Mohamadi et al. (6). Furthermore, 61.8% of cases had at least one episode of *Candida* vaginitis that similar to previous study (11). Our results show that in the studied population, antibiotic treatment, diabetes and contraceptive use were linked with vulvovaginal candidiasis as described before (11, 31, 36).

Although, *C. albicans* is considered as a vaginal mycoflora and the main causative agent of vaginal candidiasis (2, 37), non-*albicans* species have increased during last decades (3, 37, 38). Besides, a report from India indicated that *C. glabrata* was isolated from 50.4% patients with vulvovaginal candidiasis (39). Most reports have shown that *C. glabrata, C. tropicalis* and *C. krusei* were accounted as the second, third and fourth common agents of disease, respectively (14, 37, 39). Though, in the study by Mohammadi et al. *C. kefyr* was reported as the third agent of fungal vulvovaginitis in Iran (27). Furthermore, some researchers reported *C. dubliniensis* as the third causative agent (7, 14). Consistent with these facts, the frequency of *C. albicans* in our study was 88.2% followed by *C. glabrata* (8.8%). *C. kefyr* is an uncommon vulvovaginal candidiasis agent that previously was reported by Hedayati et al., (8.2%) (7), Mohammadi et al. (5.8%) (27), Fornari et al. (2.5%) (35), and Alfouzan et al. (1.9%) (16). The rate of *C. kefyr* isolation in our study was nearly similar to above reports (2.9%).

Although, topical clotrimazole, nystatin and miconazole are usually prescribed for vulvovaginal candidiasis, however a single dose of oral fluconazole is more acceptable in some patients both for prophylaxis and cure. On the other hand, resistance to fluconazole among less sensitive *Candida* species (especially non-*albicans* such as, *C. glabrata, C. tropicalis* and *C. krusei*) has been increased during last decades (30, 40). Mohanty et al. have shown that 70% and 30% of vaginal isolates of *Candida* were sensitive (MIC ≤ 8 μg/ml) and dose dependent (MIC, 16–32 μg/ml) to fluconazole, respectively and 77.8% of dose dependent isolates were *C. glabrata* (39). On the other hand, resistant rate for fluconazole was 57.1% for *C. krusei* strain in the study by Guzel et al. (30). Previously, we reported that 34.8% of vaginal isolates of *Candida* were resistant to fluconazole *in vitro* (14).

The resistance rate to *C. albicans* was 4.7% for caspofungin in vaginal isolates in China (8). In a clinical trial, no significant difference was observed in response of patients with *Candida* vaginitis after treatment with fluconazole and clotrimazole. On the other hand, disease recurrence was 6.9% and 9.7% for fluconazole and clotrimazole groups, respectively (41). In our study, all isolates were highly sensitive to fluconazole as MIC for 73.5% of them including all non-*albicans* was 0.25 μg/ml. Although, resistant to antifungal is not common, it increased during several last decades due to widespread prophylaxis use as well as to be over counter some antifungals. Furthermore, some strains of *C. krusei* and *C. glabrata* are genetically resistant to some antifungals. Diaz et al. and Moges et al. studies shown that 6 and 4 isolates of *Candida* species were respectively resistant to clotrimazole (42, 43). Balashov et al. have believe that resistance to caspofungin is linked to mutations in *FKS1* gene (44). In our study, only one isolate of *C. albicans* had MIC 2 μg/ml (Resistant) for caspofungin after 24h incubation that increased into two strains after 48h. Whereas, resistant to clotrimazole (MIC = 1μg/ml) was found in one and three isolates of *C. albicans* after 24h and 48h incubation.

## CONCLUSION

In conclusion, 28.3% of examined patients had positive cultures for three different species of *Candida*. The most common agent was *C. albicans* followed by, *C. glabrata* and *C. kefyr*. Although all of *Candida* isolates were susceptible to fluconazole *in vitro*, it should be used with caution for empirical therapy due to more resistant rates in clinic. In addition, due to valuable sensitivity of all tested strains to caspofungin, it can be presented as first line therapy for *Candida* vaginitis. However, there is not a suitable caspofungin formulation for the treatment of vaginal candidiasis due to lack of data about effect of drug on causative agents. According to our study, we can claim that caspofungin has an effective efficacy against vaginal agents. However, several clinical trials are needed for confirm our hypothesis.
